# Paratesticular cellular angiofibroma: a case report

**DOI:** 10.1186/s13256-024-04499-y

**Published:** 2024-04-11

**Authors:** Takaya Murashima, Kazutaka Kida, Toshihiro Gi, Takuya Hida, Masato Fujii, Takahiro Nagai, Hiroki Takamori, Shoichiro Mukai, Yuichiro Sato, Toshiyuki Kamoto

**Affiliations:** 1 Department of Urology, Faculty of Medicine, Miyazaki University Hospital, 5200 Kihara, Kiyotake, Miyazaki, 889-1692 Japan; 2 Department of Pathology, Faculty of Medicine, University of Miyazaki, Miyazaki, Japan; 3 Department of Diagnostic Pathology, Faculty of Medicine, Miyazaki University Hospital, Miyazaki, Japan; 4 Section of Urology, Harasanshin Hospital, Fukuoka, Japan

**Keywords:** Cellular angiofibroma, Paratesticular region, Orchiectomy

## Abstract

**Introduction:**

Paratesticular cellular angiofibroma is a rare benign mesenchymal tumor. The optimal management is surgical resection due to the difficulty of preoperative accurate diagnosis.

**Case presentation:**

A 51-year-old Japanese male visited our hospital complaining of asymptomatic left scrotal swelling. Physical examination revealed a nontender elastic paratesticular mass (5.5 cm in diameter). Although testicular germ cell tumor was ruled out clinically, the possibility of malignant potential remained for the tumor. Since the patient consented to complete resection, a transinguinal radical orchiectomy was performed. The pathological diagnosis revealed cellular angiofibroma. The patient recovered without perioperative complications, and no apparent recurrence was observed at 5 years after surgery.

**Conclusion:**

The pathological findings were compatible for cellular angiofibroma. The tumor was successfully resected, and no apparent recurrence was observed at 5 years after surgery.

## Introduction

Cellular angiofibroma is a rare benign mesenchymal tumor arising in subcutaneous tissue of the inguinoscrotal and vulvoperineal region of both genders, first described in 1997 by Nucci *et al*. [1]. The tumor has been reported to arise in the female vulvoperineal region and male inguinoscrotal region in various sizes (size range is 0.6–25 cm in females and 2.5–25 cm in males) [2]. The median age at resection is reported as 47 years in females and 60 years in males [2]. The tumor usually presents as a well-demarcated and painless mass. Optimal management in conventional cases involves complete resection. Here we report a case of cellular angiofibroma arising in the paratesticular region.

## Case presentation

**Fig. 1:**
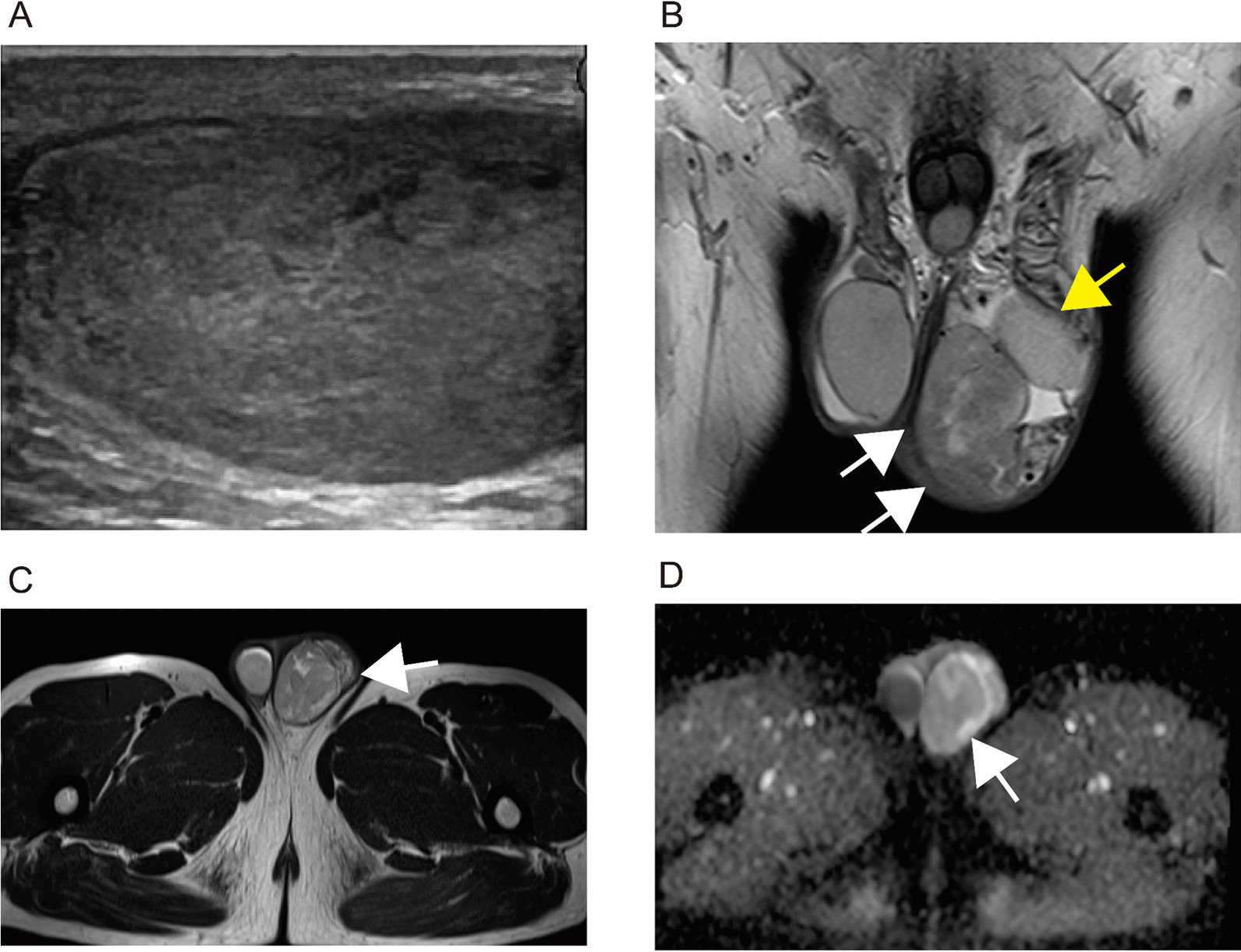
Ultrasound and magnetic resonance imaging. **A** Ultrasonography showed internally hypoechoic heterogeneous mass, 5.5-cm-long diameter in size. The tumor showed a clearly demarcated solid mass. **B**, **C** T2 weighted images by magnetic resonance imaging examination revealed heterogeneous low intensity mass (white arrows) including focal high intensity areas (**B** coronal imaging, **C** axial imaging). Left testis was confirmed (yellow arrow). **D** Diffusion-weighted imaging shows no apparent decreasing ADC value in the internal area of the mass; however, this is focally observed in peripheral area (white arrow)

A 51-year-old Japanese male visited our hospital complaining of asymptomatic left scrotal swelling. The patient had no prior history of trauma and genitourinary disease, except for benign prostatic hyperplasia. And no familial history of testicular tumor was recorded. Physical examination revealed a nontender elastic paratesticular mass. Ultrasound (US) examination revealed a clearly demarcated 5.5 cm internally heterogeneous solid paratesticular mass (Fig. 1A). T2 weighted images (T2WI) by magnetic resonance imaging (MRI) examination revealed a heterogeneous low intensity mass, including focal high intensity areas (Fig. 1A, B). Diffusion-weighted imaging (DWI) showed no apparent decreasing apparent diffusion coefficient (ADC) value in the internal area of the mass; however, this was suggested in the peripheral area (Fig. 1C, arrow). The results of laboratory examination, including alpha-fetoprotein, human chorionic gonadotropin beta-subunit, and lactate dehydrogenase, were within normal limits (Table 1). Although testicular germ cell tumor was ruled out clinically, the possibility of malignant potential remained. Since the patient consented to complete resection, a transinguinal radical orchiectomy was performed. There were no specific findings of adhesion to surrounding tissue or intraoperative complications, and the patient recovered without postoperative events. In addition, no apparent recurrence was observed at 5 years after surgery.

**Table 1:** Summary of the laboratory data on admission

Complete blood count	
White blood cells	5.7 × 10^3^/μL (3.3–8.6 × 10^3^/μL)
Neutrophils	52.8% (37–72%)
Hemoglobin	14.5 g/dL (13.7–16.8 g/dL)
Hematocrit	44.3% (40.7–50.1%)
Mean cell volume platelets	94.9 fL (83.6–98.2 fL)
Platelet counts	246 × 10^3^/μL (158–348 × 10^3^/μL)
Coagulation	
PT-INR	0.92
APTT	26.6 s (25–35 s)
Biochemistry	
Total bilirubin	0.7 mg/dL (0.4–1.5 mg/dL)
Asparate aminotransferase	22 U/L (13–30 U/L)
Alanine aminotransferase	31 U/L (10–42 U/L)
Lactate dehydrogenase	162 U/L (124–222 U/L)
γ-Glutamyl transpeptidase	38 U/L (13–64 U/L)
Alkaline phosphatase	203 U/L (106–322 U/L)
Blood-uria-nitrogen	13.8 mg/dL (8–20 mg/dL)
Creatinine	0.94 mg/dL (0.65–1.07 mg/dL)
Total protein	6.96 g/dL (6.6–8.1 g/dL)
Albumin	4.43 g/dL (4.1–5.1 g/dL)
Na	142 mmol/L (138–145 mmol/L)
K	4.5 mmol/L (3.6–4.8 mmol/L)
Cl	104 mmol/L (101–108 mmol/L)
Glucose	104 mg/dL (73–109 mg/dL)
HbA1c	5.6% (4.9–6%)
C-reactive protein	0.05 mg/dL (0–0.14 mg/dL)
HBs-Ag	(–)
HCV-Ab	(–)
Tumor marker	
sIL-2R	251 U/mL (122–496 U/mL)
AFP	4.7 ng/mL (0.89–8.78 ng/mL)
βHCG	1.2 mIU/mL (− 5mIU/L)

*PT-INR* Prothrombin Time-International Normalized Ratio; *Na* Sodium; *APTT* Activated Partial Thromboplastin Time; *K* Potassium; *Cl* Chlorine; *HbA1c* HemoglobinA1c; *HBs-Ag* hepatitis B virus antigen; *HCV-Ab* hepatitis C virus antibodt; *sIL-2R* soluble interleukin-2 receptor; *AFP* alphafetoprotein; *βHCG* human chorionic gonadotropin beta-subunit

**Fig. 2:**
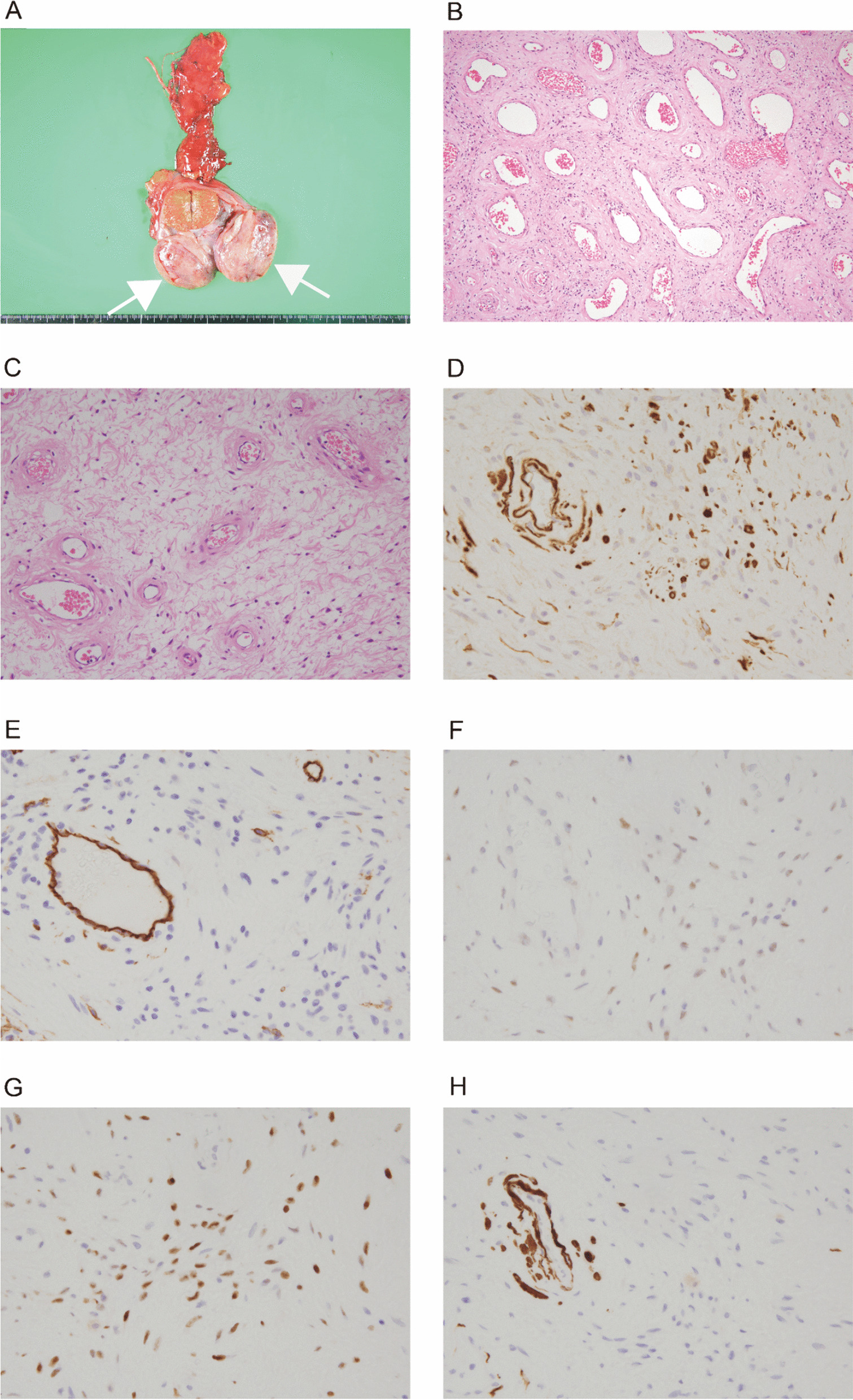
Gross and histopathological findings of the tumor. **A** Gross findings. The paratesticular tumor (white arrows) is well-circumscribed, detached from the testis (**A**). Cut surface shows heterogeneous greyish-white in color. **B** Microscopically, tumor lesion shows proliferation of short spindle cells in a diffuse or perivascular pattern, accompanied by collagenous stromal fibers and dilated vascular channels. No nuclear atypia or mitotic figure is shown. **C** Myoid stromal areas are focally seen with hypocellularity. **D**–**H** Immunohistochemical findings. Short spindle cells are positive for alpha-smooth muscle actin (**D**), CD34 (**E**), estrogen receptor (**F**), and progesterone receptor (**G**) and negative for desmin (**H**). Vascular walls and endothelial cells are also positive for alpha smooth muscle actin and CD34 and desmin, respectively

On gross examination, the tumor measured 5.5 cm and was heterogeneous grayish-white in color. The tumor was encapsulated by fibrous tissue and detached from the testis (Fig. 2A). Microscopically, the tumor showed mixed hypercellular and hypocellular regions. The former showed proliferation of fibroblastic short spindle cells in a diffuse or perivascular pattern accompanied by collagenous fibers and dilated vascular channels (Fig. 2B). In the latter, some myoid stromal areas were seen with a few fibroblastic cells (Fig. 2C). Immunohistochemically, short spindle cells were positive for alpha-smooth muscle actin (SMA), CD34, estrogen receptor, and progesterone receptor and negative for desmin (Fig. 2D–H). The location and histopathological findings were compatible with the diagnosis of cellular angiofibroma.

## Discussion

Cellular angiofibroma associated with morphological overlap with spindle cell lipoma, angiomyofibroblastoma, and aggressive angiomyxoma has been reported [2–4]. Compared with cellular angiofibroma, occurrence in the genital area is uncommon in spindle cell lipoma. Microscopically, the tumor is mainly composed of adipocytes with ropy collagen, and vasculature is not pronounced [2–4]. In addition, the appearance of hyalinized thick-walled vessels is not seen in spindle cell lipoma. The spindle cells have strong immunoreactivity for CD34 against cellular angiofibroma [3, 4].

Angiomyofibroblastoma shows a circumscribed and usually encapsulated appearance, predominantly occurring in young to middle-aged female genital tract [4]. In contrast to cellular angiofibroma, cellular (spindle and epithelioid) and nuclear (mononucleate and multinucleate) polymorphism is distinctive [3, 4]. A strong tendency for perivascular aggregation of tumor cells and the presence of numerous delicate capillary-sized vessels are also reported as characteristics [3, 4].

Aggressive angiomyxoma is a poorly demarcated tumor, usually occurring in the pelvic soft tissue and perineal region of young females (occurrence in males is rare). Pathologically, the cellularity and vascularity are lower than in cellular angiofibroma, and myxedematous background is more evident in aggressive angiomyxoma [3, 4]. In addition, the tumor cells show strong immunoreactivity for desmin compared with cellular angiofibroma [3].

As mentioned above, a diagnosis of cellular angiofibroma is generally based on morphological and architectural findings without specific immunohistochemical markers.

However, monoallelic 13q14 deletion has been reported in cellular angiofibroma, and the downregulation of *RB1* and *FOXO1* (both genes are encoded in 13q14) was observed, suggesting these as promising significant markers [5].

As for prognosis in cellular angiofibroma, no metastases have been recorded [6–9]. However, a few cases of local recurrence have been reported. Therefore, pathological diagnosis of surgical margin should be carefully considered, and postoperative follow-up may be necessary [9]. The appearance of scrotal mass in analysis with magnetic MRI has been discussed in several reports [8, 9]. Cellular angiofibroma is usually revealed as a heterogeneous high signal intensity mass on T2WI, and intense heterogeneous enhancement has been observed by gadolinium administration [8, 9]. In addition, DWI usually shows the absence of restricted diffusion [8, 9]. The majority of T2WI and DWI appearance in our case was consistent with that of previous reports (despite not administering gadolinium in the current case). As paratesticular tumors, exclusion of malignant tumors, including sarcomas (liposarcoma, leiomyosarcoma, and rhabdomyosarcoma) and paratesticular metastasis, was necessary. The presence of diffusion restriction and initial upstroke with subsequent gradual washout on dynamic contrast MRI were reported as specific findings in these malignant tumors [8, 9].

## Conclusion

We report a case of paratesticular cellular angiofibroma. Since the possibility of malignant potential remained, transinguinal radical orchiectomy was performed. The pathological findings were compatible with ordinary cellular angiofibroma. The patient recovered without perioperative complications and no apparent recurrence was observed at 5 years after surgery.

## Data Availability

The supporting data and materials for this report are available on request from corresponding author.

## Abbreviations

US: Ultrasound
T2WI: T2 weighted image
MRI: Magnetic resonance imaging
DWI: Diffusion-weighted imaging
ADC: Apparent diffusion coefficient
SMA: Alpha-smooth muscle actin
CD34: Cluster of differentiation 34
